# Polygenic risk for mental disorders as predictors of posttraumatic stress disorder after mild traumatic brain injury

**DOI:** 10.1038/s41398-023-02313-9

**Published:** 2023-01-25

**Authors:** Murray B. Stein, Sonia Jain, Livia Parodi, Karmel W. Choi, Adam X. Maihofer, Lindsay D. Nelson, Pratik Mukherjee, Xiaoying Sun, Feng He, David O. Okonkwo, Joseph T. Giacino, Frederick K. Korley, Mary J. Vassar, Claudia S. Robertson, Michael A. McCrea, Nancy Temkin, Amy J. Markowitz, Ramon Diaz-Arrastia, Jonathan Rosand, Geoffrey T. Manley, TRACK-TBI Investigators, Neeraj Badjatia, Ann-Christine Duhaime, Adam R. Ferguson, Shankar Gopinath, Ramesh Grandhi, Christopher Madden, Randall Merchant, David Schnyer, Sabrina R. Taylor, John K. Yue, Ross Zafonte

**Affiliations:** 1 Department of Psychiatry, University of California, San Diego, La Jolla, CA USA; 2 School of Public Health, University of California, San Diego, La Jolla, CA USA; 3 VA San Diego Healthcare System, San Diego, CA USA; 4 Biostatistics Research Center, Herbert Wertheim School of Public Health and Human Longevity Science, University of California, San Diego, La Jolla, CA USA; 5 Center for Genomic Medicine, Massachusetts General Hospital, Boston, MA USA; 6 McCance Center for Brain Health, Massachusetts General Hospital, Boston, MA USA; 7 Broad Institute of MIT and Harvard, Cambridge, MA USA; 8 Department of Psychiatry, Massachusetts General Hospital, Boston, MA USA; 9 Departments of Neurosurgery and Neurology, Medical College of Wisconsin, Milwaukee, WI USA; 10 Department of Radiology & Biomedical Imaging, UCSF, San Francisco, CA USA; 11 Department of Bioengineering & Therapeutic Sciences, UCSF, San Francisco, CA USA; 12 Department of Neurological Surgery, University of Pittsburgh Medical Center, Pittsburgh, PA USA; 13 Department of Physical Medicine and Rehabilitation, Harvard Medical School, Boston, MA USA; 14 Spaulding Rehabilitation Hospital, Charlestown, MA USA; 15 Department of Emergency Medicine, University of Michigan, Ann Arbor, MI USA; 16 Brain and Spinal Cord Injury Center, Zuckerberg San Francisco General Hospital and Trauma Center, San Francisco, CA USA; 17 Department of Neurological Surgery, UCSF, San Francisco, CA USA; 18 Department of Neurosurgery, Baylor College of Medicine, Houston, TX USA; 19 Departments of Neurological Surgery and Biostatistics, University of Washington, Seattle, WA USA; 20 Department of Neurology, University of Pennsylvania, Philadelphia, PA USA; 21 University of Maryland, College Park, MD USA; 22 MassGeneral Hospital for Children, Boston, MA USA; 23 University of Utah, Salt Lake City, UT USA; 24 UT Southwestern, Dallas, TX USA; 25 Virginia Commonwealth University, Richmond, VA USA; 26 UT Austin, Austin, TX USA

**Keywords:** Biomarkers, Psychiatric disorders

## Abstract

Many patients with mild traumatic brain injury (mTBI) are at risk for mental health problems such as posttraumatic stress disorder (PTSD). The objective of this study was to determine whether the polygenic risk for PTSD (or for related mental health disorders or traits including major depressive disorder [MDD] and neuroticism [NEU]) was associated with an increased likelihood of PTSD in the aftermath of mTBI. We used data from individuals of European ancestry with mTBI enrolled in TRACK-TBI (*n* = 714), a prospective longitudinal study of level 1 trauma center patients. One hundred and sixteen mTBI patients (16.3%) had probable PTSD (PCL-5 score ≥33) at 6 months post-injury. We used summary statistics from recent GWAS studies of PTSD, MDD, and NEU to generate polygenic risk scores (PRS) for individuals in our sample. A multivariable model that included age, sex, pre-injury history of mental disorder, and cause of injury explained 7% of the variance in the PTSD outcome; the addition of the PTSD-PRS (and five ancestral principal components) significantly increased the variance explained to 11%. The adjusted odds of PTSD in the uppermost PTSD-PRS quintile was nearly four times higher (aOR = 3.71, 95% CI 1.80–7.65) than in the lowest PTSD-PRS quintile. There was no evidence of a statistically significant interaction between PTSD-PRS and prior history of mental disorder, indicating that PTSD-PRS had similar predictive utility among those with and without pre-injury psychiatric illness. When added to the model, neither MDD-PRS nor NEU-PRS were significantly associated with the PTSD outcome. These findings show that the risk for PTSD in the context of mTBI is, in part, genetically influenced. They also raise the possibility that an individual’s PRS could be clinically actionable if used—possibly with other non-genetic predictors—to signal the need for enhanced follow-up and early intervention; this precision medicine approach needs to be prospectively studied.

## Introduction

Traumatic brain injury (TBI) is increasingly being recognized as an important contributor to the global burden of disease [1, 2]. Whereas moderate-to-severe TBIs have long been known to result in morbidity and mortality, so-called “mild” TBIs have more recently come to be appreciated as medically and socioeconomically important in their own right. Many patients with mild traumatic brain injury (mTBI) do not fully recover from their injury [3], and psychological health problems such as posttraumatic stress disorder (PTSD) and major depressive disorder (MDD) frequently contribute to residual dysfunction and reduced quality of life [4–6].

PTSD is seen in upwards of 20% of patients with mTBI and more commonly than in patients with non-head orthopedic injuries [7, 8]. It has been hypothesized that the high rate of PTSD in the context of mTBI stems, at least in part, from injury to shared brain circuitry involving the prefrontal cortex, which plays an important role in emotion regulation, and the hippocampus, which subserves memory [9–12]. It is also apparent that individual differences in cognitive function and personality modify risk for mental disorders such as PTSD after mTBI [5, 13]. But little is known about the role of genetic factors in the risk for PTSD in the context of mTBI.

Genome-wide association studies (GWAS) suggest that multiple risk loci are involved in the etiology of PTSD and related disorders [14–18]. Genotypic data for variants associated with disease risk can be pooled and expressed as polygenic risk scores (PRS) that index overall genetic liability for that condition, which demonstrate greater explanatory power in predictive models of most complex disease phenotypes than single risk variants [19]. Studies have begun to evaluate if PRS can predict PTSD onset [20] and symptom profiles [21] among individuals exposed to traumatic stress.

To the best of our knowledge, no study to date has examined whether PRS for PTSD is associated with PTSD diagnosis among individuals who experience traumatic brain injury. Using data from a prospective longitudinal study of individuals seen in emergency departments for mild traumatic brain injury, we hypothesized that polygenic risk for PTSD (PTSD-PRS) would be associated with increased risk for PTSD after injury. We further hypothesized that polygenic risk for major depressive disorder (MDD-PRS) and neuroticism (NEU-PRS), two traits more broadly related to stress-related psychopathology, would also contribute to prediction of PTSD risk post-injury. If PRS is shown to be predictive for mental disorders such as PTSD in this context, consideration could be given to their future use to identify individuals at risk for PTSD after injury, thereby permitting enhanced outreach and surveillance and, if needed, early intervention.

## Participants and methods

### Overview

Transforming Research and Clinical Knowledge in TBI (TRACK-TBI) is an 18-center prospective observational study of subjects evaluated in level I trauma centers within 24 hours of injury from 2/26/2014 through 8/08/2018 [3]. Our analysis included *n* = 714 subjects of European Ancestry, age ≥ 17 years, with Glasgow Coma Scale score on hospital arrival of 13–15, enrolled between March 2014 and July 2018, had availability of PCL-5 scores at 6 months post-injury, and had been array genotyped permitting the calculation of PRS scores. Inclusion criteria for the broader study were having one’s treating physician order a head computed tomography scan due to suspicion of TBI; meeting the American Congress of Rehabilitation Medicine definition of TBI; adequate visual acuity/hearing pre-injury; and fluency in English or Spanish. Exclusion criteria included: significant polytrauma that would interfere with follow-up; penetrating TBI; prisoners or patients in custody; pregnancy; major debilitating mental (e.g., schizophrenia, bipolar disorder) or neurological disorder (e.g., stroke, dementia) or any other disorder that would interfere with assessment and follow-up; current participant in an interventional trial. Written informed consent was obtained from subjects or legally authorized representatives. The study was approved by the IRBs of enrolling sites.

### Measures

PTSD Checklist for DSM-5 (PCL-5): The PCL-5 is a widely used measure of posttraumatic stress disorder symptoms. The range of the scale is 0–80. Signal detection analyses against a clinical gold standard revealed that PCL-5 cut scores of 31 to 33 were optimally efficient for diagnosing PTSD [22]. Consistent with our prior work in this area, we used scores of ≥33 to indicate probable PTSD [8].

Glasgow Coma Scale (GCS): The GCS is a widely used estimate of brain injury severity that characterizes gross level of consciousness soon after injury (range 3–15; 13–15 is customarily considered “mild” TBI) [23].

Past Psychiatric History: The TRACK-TBI Interview requested information from the respondent (acquired at baseline, and in some cases collected from a relative or other suitable informant) about prior history of mental disorder as evidenced by prior diagnoses or treatment. For purposes of this study, these data were coded as binary, i.e., history of mental disorder vs. no history of mental disorder.

### DNA collection and genotyping

The methods for DNA collection, genotyping, imputation, quality control, and ancestry assignment are reported in a previous publication [24]. Genotyping of the TRACK-TBI individuals was conducted at the Broad Institute of MIT and Harvard, using the Illumina Global Screening Array (GSA-24v2–0 + Multi-Disease). Standard quality control procedures were applied and the array-based genotypes were imputed using the Haplotype Reference Consortium reference panel [25].

### Polygenic risk scores

We used summary statistics from recent GWAS studies of PTSD [14], MDD [26], and neuroticism [27] phenotypes to estimate SNP effect sizes for polygenic scoring. PLINK 2.0 [28] was used to calculate PRS based on the sum of all available SNPs weighted by their effect sizes adjusted for linkage disequilibrium using PRS-CS-auto [29] for each individual in the target cohort. PRS was standardized within the sample for subsequent analyses. PRS analyses were conducted only in the European ancestry subsamples because of the unavailability of reference GWAS data for other populations [30, 31].

### Statistical analysis

Demographics and clinical characteristics were summarized for the study cohort. Spearman’s correlations were calculated among the PRS. Univariate and multivariable logistic regression models assessed whether PRS were independent predictors of PTSD adjusting for known risk factors including age, sex, history of mental disorder prior to injury, and cause of injury (dichotomized as violence/assault vs. non-assaultive) [8], and five ancestral principal components (PCs). As a sensitivity analysis, we also reran these analyses with PCL-5 score at 6 months as the outcome, using multivariable linear regression. Statistical significance was set as a *p* value < 0.05. Statistical analyses were conducted in R, version 4.1.2 (R Core Team, 2013).

## Results

Of 714 patients with PCL-5 scores available at 6 months post-injury, 116 (16.3%) had probable PTSD (PCL-5 score ≥33). Mean age of patients was 44.6 (SD 18.2) years and 65% were male. Traffic accidents or fall were the most common cause of injury (97.05%) with violence or assault being relatively rare (2.95%). More than one-quarter (29.4%) of patients had a pre-injury history of mental disorders [Table 1].

**Table 1:** Demographic and clinical characteristics of the study sample (*N* = 714).

	Number (%)
Highest level of care	
ED discharge	195 (27.3%)
Hospital admit no ICU	311 (43.6%)
Hospital admit with ICU	208 (29.1%)
Total	714 (100%)
Sex	
Male	462 (64.7%)
Female	252 (35.3%)
Total	714 (100%)
GCS at admission	
13	23 (3.2%)
14	141(19.8%)
15	550 (77.0%)
Total	714 (100%)
Psychiatric history	
No	504 (70.6%)
Yes	210 (29.4%)
Total	714 (100%)
Injury cause	
Traffic incident/fall/other	691 (97.05%)
Violence/assault	21 (2.95%)
Total	712 (100%)
Age, years (Mean (SD))	44.6 (18.2)

ED emergency department, ICU intensive care unit; GCS, Glasgow Coma Scale.

The 3 PRS scores were moderately intercorrelated: PTSD-PRS and MDD-PRS, *r*_s_ = 0.44, *p* < 0.001; PTSD-PRS and NEU-PRS, *r*_s_ = 0.30, *p* < 0.001; MDD-PRS and NEU-PRS, *r*_s_ = 0.53, *p* < 0.001.

**Fig. 1:**
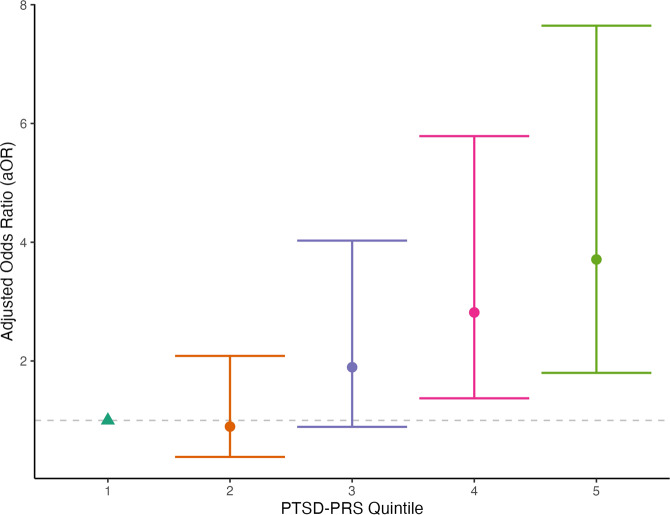
Adjusted odds ratio (and 95% CI) for 6-months PTSD by PTSD-PRS quintiles. Dots indicate the point estimate for adjusted odds ratio (aOR) and the lines emanating from the dots indicate the upper and lower 95% confidence intervals. The triangle indicated an aOR of 1 for the lowest quintile, which is the category to which the other quintiles are compared.

The PRS for PTSD (PTSD-PRS) alone, adjusting for 5 PCs, was associated with increased odds of PTSD (aOR = 1.82, 95% CI 1.45–2.27 per standard unit increase in PTSD-PRS), explaining ~7% of the variance in the 6-month PTSD outcome. A multivariable model that included age, sex, pre-injury history of mental disorder, and cause of injury explained ~5% of the variance in the 6-month PTSD outcome (Table 2a, Model 1). A model that combined both sets of predictors (Table 2a, Model 2) significantly outperformed Model 1 (Delong’s test *p-value* = 0.023 comparing AUC of Model 2 vs. Model 1, LRT *p* < 0.001 comparing Model 2 to Model 1), explaining 11.3% of the variance in the 6-month PTSD outcome. The adjusted odds of PTSD in the uppermost PTSD-PRS quintile were nearly four times higher (aOR = 3.71, 95% CI 1.80–7.65) than in the lowest PTSD-PRS quintile (Fig. 1). There was no evidence of a statistically significant interaction between PTSD-PRS and prior history of mental disorder (*p* = 0.82), indicating that PTSD-PRS predicted 6-month PTSD similarly among those with and without pre-injury psychiatric illness. Similarly, there were no statistically significant interactions between PTSD-PRS and age (*p* = 0.90), sex (*p* = 0.86), or injury cause (*p* = 0.64).

**Table 2:** Multivariable logistic regression models assessing the association between various risk factors and probable PTSD at 6 months post-injury.

a Multivariable logistic regression model assessing the association between PTSD polygenic risk score (PTSD-PRS) and odds of probable PTSD at 6 months post-injury.					
MODEL 1 (without PTSD-PRS)	OR	95% CI lower	95% CI upper	Wald Chi-square	*p* value
Age (years)	0.99	0.974	0.998	5.23	0.022
Sex female vs male	1.13	0.733	1.74	0.31	0.579
Psychiatric history	3.14	2.06	4.79	28.2	<0.0005
Injury cause (violence/assault vs. traffic/fall/other)	2.37	0.89	6.32	2.99	0.084
MODEL 1 (without PTSD-PRS) PRS, Pseudo-Rsq = 0.07, AUC = 0.687					

MODEL 2 (with PTSD-PRS)*	OR	95% CI lower	95% CI upper	Wald Chi-square	*p* value
Age (years)	0.99	0.975	1.00	4.13	0.042
Sex female vs male	1.20	0.767	1.86	0.62	0.431
Psychiatric history	3.02	1.95	4.66	24.66	<0.0005
Injury cause (violence/assault vs. traffic/fall/other)	3.09	1.12	8.53	4.75	0.029
PTSD-PRS	1.77	1.40	2.23	23.10	<0.0005
MODEL 1 (with PTSD-PTSD) PRS, Pseudo-Rsq - 0.113, AUC = 0.733. Delong’s test p = 0.023 comparing AUC of Model 2 vs. Model 1, LRT *p* < 0.001					

b Multivariable logistic regression model assessing the association between polygenic risk scores for PTSD (PTSD-PRS) and MDD (MDD-PRS) and odds of probable PTSD at 6 months post-injury*					
	OR	95% CI lower	95% CI upper	Wald Chi-square	*p* value
Age (years)	0.987	0.975	1.00	3.97	0.046
Sex female vs male	1.20	0.77	1.88	0.66	0.417
Psychiatric history	2.97	1.92	4.59	23.75	<0.0005
Injury cause (violence/assault vs. traffic/fall/other)	3.14	1.13	8.70	4.84	0.028
PTSD-PRS	1.680	1.31	2.16	16.42	<0.0005
MDD-PRS	1.13	0.89	1.43	1.04	0.308

*Also simultaneously included in the multivariable model, but not shown, are the first five ancestral principal components.

A sensitivity analysis was undertaken to determine if the PTSD-PRS made a similar contribution to explaining variance in the PTSD outcome when that outcome was considered as a continuous measure, using the PCL-5 score at 6 months. Compared to a model that included age, sex, pre-injury history of mental disorder, and cause of injury—which explained 9.7% of the variance in 6-month PTSD severity—the addition of the PTSD-PRS and the five PCs significantly improved the variance explained to 13.4% (LRT *p* < 0.001) [see Supplementary Tables 1, 2].

NEU-PRS (adjusted for five PCs) was not significantly associated with PTSD at 6 months in an identical model that included age, sex, pre-injury history of mental disorder, and injury cause (aOR = 1.06, 95% CI 0.86–1.31, *p* = 0.59). MDD-PRS, on the other hand, *was* significantly associated with PTSD in an identical model (aOR = 1.37, 95% CI 1.11–1.69). However, when added to a model that simultaneously included PTSD-PRS, MDD-PRS was no longer significantly associated with the PTSD outcome (aOR = 1.13, 95% CI 0.89–1.43) [Table 2b].

## Discussion

Traumatic brain injury represents a significant health crisis in the United States and worldwide. The majority of TBIs are classified as mild (GCS 13–15) [32]. Although most individuals who sustain a mild TBI will go on to recover completely, up to 20% will suffer from psychiatric illness such as PTSD, particularly in the first 6 months post-injury [5, 8, 33]. With finite healthcare resources available, the ability to predict clinical outcomes to allocate resources toward individuals at the greatest risk of developing chronic post-TBI symptoms and disability could lead to both cost-savings and improvement in individual quality of life. This is particularly true for costly and impairing sequelae such as PTSD [34], for which proven treatments, including early psychosocial interventions, exist. In fact, implementing clinical intervention as soon as possible following a traumatic event leads to a decreased likelihood of developing PTSD [35].

Previous studies have indicated that certain demographic features such as age, sex, prior mental illness, and cause of injury are associated with a differential risk of developing PTSD following TBI [8, 36]. In this study, we adjusted for those factors and determined whether one or several PRS for neuroticism (a general risk factor for psychopathology), major depressive disorder (a common mental disorder frequently comorbid with PTSD), and PTSD, per se, were associated with PTSD 6 months following mild TBI. Since genetic susceptibility is shared across mental disorders [37], and PRS can be predictive across diagnostic categories [20, 38], we examined several mental health-related PRS. To the best of our knowledge, this study is the first to test whether PRS for PTSD or other mental health-related PRS is associated with an increased risk for PTSD following physical (in this case, brain) injury.

We found that PRS-PTSD score was significantly associated with the presence and severity of PTSD 6 months following mTBI, adding substantially to the predictive power of models that took into account (i.e., adjusted for) other pre-injury risk factors including age, sex, pre-injury history of mental illness, and cause of injury. These findings show that the risk for PTSD in the context of mTBI is, in part, genetically mediated, likely through genetic factors associated with PTSD risk in general. Individuals in the highest quintile of PTSD-PRS had nearly four times the odds of PTSD than those in the lowest quintile, indicating that PTSD-PRS rivals or exceeds in predictive capacity many other oft-replicated pre-trauma risk factors for PTSD (e.g., psychiatric history or cause of injury) [39]. Accordingly, they also raise the possibility that an individual’s PTSD-PRS could be clinically actionable if used to signal the need for enhanced follow-up and possible early intervention. As noted above, given the fact that evidence-based early interventions for PTSD have been shown to reduce morbidity [35], the use of indicators such as PTSD-PRS could potentially facilitate the targeting of prevention efforts within high-risk strata.

Studies of the latent structure of mental disorders consistently find that PTSD and MDD load together on a Distress Disorders subfactor of Internalizing Disorders [40], suggesting a high degree of shared vulnerability to these two disorders. Consistent with this observation, although PTSD-PRS was the strongest polygenic predictor of PTSD at 6 months post-injury, MDD-PRS also had reasonable predictive power for PTSD (whereas NEU-PRS did not). Several studies provide data that may help us understand this finding. Coleman et al. found that the genetic contribution to MDD was greater when reported trauma was present [41]. Although not directly addressing PTSD, that finding could indicate that genetic risks for MDD and PTSD converge in the presence of traumatic events. Another study more directly addressed the overlap of genetic risk for MDD and PTSD, where findings pointed to the existence of genetic variants associated with trauma sensitivity that might be shared between PTSD and MDD [42]. It remains to be determined to what extent polygenic risks for PTSD and MDD are shared in different stress and injury exposure contexts, and whether differential prediction will be possible.

Strengths of this study include its multi-center, longitudinal, prospective design, the large number of participants, and the use of multivariable statistical analysis that incorporated non-genetic predictors in addition to PRS. However, this study also has limitations. It was limited to adults and adolescents age 17 and older presenting to level 1 trauma centers who required a head CT scan, and had 6-month follow-up assessments. Individuals who did not have 6-month follow-up assessments might be more (or less) ill, and PRS prediction might have differed if data from those individuals had been available for analysis. This study also relied on self-reports of prior history of psychiatric illness, which could lead to recall and reporting biases. Although the PCL-5 is a standardized assessment with good validity for making provisional PTSD diagnoses [22], an interview by an experienced clinician remains the gold standard for diagnosis. As noted above, the study was also limited by the availability of external PRS only for individuals of European ancestry; expansion into other ancestral groups is a priority going forward [43].

## Conclusions

This study showed that the risk for PTSD following mTBI has, in part, a genetic basis and that a polygenic risk score for PTSD can be used to stratify individuals into those at higher and lower risk. It is conceivable that PTSD-PRS (or future iterations of PRS with even better predictive power) could be incorporated into cost-effective methods for estimating risk [19] and facilitate targeting of prevention or early intervention efforts to those at the highest risk. This hypothesis remains to be tested in future trials.

## Supplementary information

Supplementary Tables
