# Supplementary material for: Polygenic risk for mental disorders as predictors of posttraumatic stress disorder after mild traumatic brain injury
Source: Transl Psychiatry. 2023 Jan 25;13:24. doi: 10.1038/s41398-023-02313-9 (PMC9873804; doi:10.1038/s41398-023-02313-9)
Supplement: Supplementary file 1 — Supplementary Tables [file 41398_2023_2313_MOESM1_ESM.pdf]

Supplementary Table 1: n=712

	Est	Est.lower	Est.upper	Chisq	pvalue
Age (years)	−0.110	−0.174	−0.046	11.47	0.001
Sex Female vs. Male	2.907	0.447	5.367	5.36	0.021
Psychiatric History	8.511	5.941	11.080	42.14	<0.0005
Injury Cause	9.249	2.426	16.071	7.06	0.008

Rsq=0.097

Supplementary Table 2: n=712

	Est	Est.lower	Est.upper	Chisq	pvalue
PTSD-PRS	3.227	2.022	4.432	27.55	<0.0005
Age (years)	−0.094	−0.157	−0.031	8.52	0.004
Sex Female vs. Male	3.146	0.718	5.574	6.45	0.011
Psychiatric History	8.016	5.465	10.568	37.91	<0.0005
Injury	10.431	3.679	17.184	9.17	0.002
PC1	0.923	−1.253	3.100	0.69	0.406
PC2	0.554	−0.973	2.082	0.51	0.477
PC3	−0.165	−0.801	0.472	0.26	0.612
PC4	−0.075	−0.710	0.559	0.05	0.816
PC5	−0.294	−0.800	0.212	1.30	0.255

Rsq=0.134, LRT.pval<0.001 compared to Table 1 model.
